# Clinical pharmacist-led assessment and management of anticholinergic burden and fall risk in geriatric patients

**DOI:** 10.1186/s12877-023-04599-2

**Published:** 2023-12-15

**Authors:** Hilal Gökçay Saz, Nadir Yalçın, Kutay Demirkan, Meltem Gülhan Halil

**Affiliations:** 1 Department of Clinical Pharmacy, Faculty of Pharmacy, Hacettepe University, Ankara, 06230 Turkey; 2 Division of Geriatric Medicine, Department of Internal Medine, Faculty of Medicine, Hacettepe University, Ankara, 06230 Turkey

**Keywords:** Anticholinergic burden, Aachen Falls Prevention Scale, Clinical pharmacist, Fall risk, Intervention

## Abstract

**Background:**

The aim of this study was to examine the risk of fall with the surrogate outcome of the Aachen Falls Prevention Scale and to assess the clinical pharmacist interventions in order to minimize anticholinergic drug burden and associated risk of fall according to a fall risk assessment scale in the older adults.

**Methods:**

Patients who admitted to the geriatric outpatient clinic of a university hospital and taking at least one anticholinergic drug were evaluated both retrospectively and prospectively as groups of different patients by the clinical pharmacist. Patients’ anticholinergic burden was assessed using the Anticholinergic Cognitive Burden Scale. For fall risk assessment, the Aachen Falls Prevention Scale was also administered to each patient whose anticholinergic burden was determined in the prospective phase of the study.

**Results:**

A total of 601 patients were included. Risk of falls increased 2.50 times in patients with high anticholinergic burden (OR (95% CI) = 2.503 (1.071–5.852); *p* = 0.034), and the existing history of falls increased the risk of high anticholinergic burden 2.02 times (OR (95%CI) = 2.026 (1.059–3.876); *p* = 0.033). In addition, each unit increase in the fall scale score in the prospective phase increased the risk of high anticholinergic burden by 22% (*p* = 0.028). Anticholinergic burden was significantly reduced as a result of interventions by the clinical pharmacist in the prospective phase (*p* = 0.010).

**Conclusion:**

Our study revealed that incorporating a clinical pharmacist in the handling of geriatric patients aids in the detection, reduction, and prevention of anticholinergic adverse effects.

## Background

Anticholinergic drugs consist of different pharmacological groups which are frequently used varies between 8 and 37% in older adults [1, 2]. These drugs are widely used by older adults for various conditions including overactive bladder, seasonal rhinitis, depression, pain, and insomnia [3, 4].

Anticholinergic drugs act on muscarinic receptors in the central and peripheral nervous systems and inhibit acetylcholine-mediated effects by binding competitively to these receptors [5]. Medications with anticholinergic activity predominantly affect muscarinic receptors [6]. M1 and M2 receptor activation, which constitute the majority of the total muscarinic receptors in the human body, is more important for cognitive processes [7]. Due to this mechanism, the broad presence of drugs with anticholinergic effects increases cognitive side effects. Due to age-related changes in pharmacokinetic and pharmacodynamic effects, decreased acetylcholine-mediated transmission in the brain, and increased permeability of the blood-brain barrier, older adults may become more sensitive to anticholinergic side effects [8, 9]. It is anticipated that the therapeutic effects expected in the clinic may be compromised by the risk of these adverse effects that may occur as a result of extensive usage of these medications [10]. Although the results are controversial, anticholinergic drug use in the older adults has been linked to a higher risk of dementia, delirium, falls, hospitalization, urinary retention, constipation, dry mouth, confusion, delirium, decreased cognitive abilities, as well as mortality [11–13]. Anticholinergic burden (ACB), on the other hand, has been identified as a substantial independent risk factor for anticholinergic consequences [14]. As a result, anticholinergic medications are generally regarded as inappropriate for older persons [15]. Furthermore, the combination of multiple medications with lower anticholinergic activity may exacerbate these medications’ anticholinergic effects [16].

Expert opinion derived these risk scales are routinely used in research and clinical practice to quantify ACB [17] Rating scales informed by expert opinions typically categorize the anticholinergic activity of pharmaceuticals into four tiers, spanning from an absence of recognized anticholinergic activity (designated as 0) to a distinct or high level of anticholinergic activity (designated as 3) [18]. Due to the inclusion of more than one drug class in the classification of anticholinergic medicines, as well as variances in anticholinergic effects of pharmaceuticals, multiple scales and methods for measuring ACB have been devised [16]. These scales consist of drug lists or equations that categorize and score pharmaceuticals based on their anticholinergic action. The ACB is represented by the sum of these anticholinergic scores, and it can be predicted that the probability of anticholinergic adverse effects increases as the score increases [19].

The aim of this study was to examine the effect of the anticholinergic burden on the falls and to assess the clinical pharmacist interventions in order to minimize anticholinergic drug burden and associated risk of fall in the older adults.

## Methods

### Study participants

This study, which consisted of retrospective (January-May 2021) and prospective (May-September 2021) process, was conducted in the geriatric outpatient clinic of a university hospital providing tertiary health care. The retrospective (control group) process was a cross-sectional study of admitted patients, whereas the prospective (intervention group) process was a cohort study with a clinical pharmacist intervention for prevention. Patients included in the retrospective data file were not included in the prospective process.

Patients aged 65 years and older who were routinely followed up, who used at least 1 anticholinergic drug and agreed to participate in the study, and whose participation in the study was deemed appropriate by the responsible geriatric physician were included in the study. Patients refused to give written consent, had impaired cognitive function, and had difficulty in communication were excluded from the study.

### Study variables

ACB of the patients using at least one anticholinergic drug was determined by clinical pharmacist with the Anticholinergic Cognitive Burden Scale (ACBS). Based on the current literature, the cut-off value is the ≥ 3 points [20]. It outperforms other scales in demonstrating anticholinergic burden in older adults, providing a dose-response relationship for side effects, and determining anticholinergic medicines. It aids in the fast identification of medicines linked with cognitive function impairment and the optimization of clinical decisions [21]. The updated version in 2012 after a review of new information and newly approved medications, was used in this study [22].

In the retrospective process, each patient’s ACB was assessed once, while in the prospective process it was assessed before and after the intervention to determine the impact of the clinical pharmacist in ACB. Demographic data of the patients (age, gender, marital status, height-weight, smoking, alcohol use, allergy status, vaccination information, history of a fall within the last year), medications, laboratory findings, Charlson Comorbidity Index (CCI), geriatric screening tests [(Activities of Daily Living (ADLs), Instrumental Activities of Daily Living (IADLs), Mini Nutritional Assessment-Short Form (MNA-SF), Geriatric Depression Scale (GDS), Mini Mental State Examination (MMSE), and Clock Drawing Test (CDT)] applied to patients by their geriatricians in outpatient clinic routine were obtained from the electronic health records and patients. Subsequently, the Aachen Falls Prevention Scale (AFPS) was administered to patients with ACB in the prospective process of the study to assess the risk of falls. The AFPS is a 3-step multifactorial and functional assessment. In the first step, it consists of 10 standard “yes/no” questions including typical risk factors such as cognitive or visual impairment, presence of incontinence, history of falls, inappropriate footwear or any items at home that may cause falls, health problems that may increase the risk of falls (osteoporosis, Parkinsonism, arthritis, rheumatic diseases) and medications used at home. Patients are considered to be at high risk for falls if they score 5 points or more in this step. The second step is a standing test without any corrective movement for 20 s to determine balance problems. Finally, the third step consists of a 10-point Likert scale in which patients subjectively evaluate their own fall risk [23].

Following the clinical pharmacist’s assessment of medications and adverse effects, physicians were contacted if an anticholinergic intervention was required and these recommendations were also recorded.

### Statistical analysis

A small effect size was predicted according to Cohen’s d statistic (G*Power Version 3.0.10). Therefore, it was planned to include a total of 580 patients in the study, 290 patients each retrospectively and prospectively, with an effect size of 0.30, 95% power and 5% margin of error, based on the study by Naharci et al. [24].

As descriptive statistics, mean and standard deviation or median and range for numerical variables and number and percentage values for categorical variables were given. Normality assumption, was analyzed by Kolmogorov-Smirnov test and graphical representations. In the comparison of numerical data, student’s t-test was used for normally distributed data and Mann-Whitney U test was used for non-normally distributed data. Chi-Square test was used to compare the ratios. In analyzing the change over time, the significance test of the difference between two pairs or Wilcoxon test was used. The relationship between numerical variables was analyzed using the appropriate correlation test (Pearson or Spearman). Phi and Cramer’s V coefficients were used to evaluate whether the statistically significant difference (*p* < 0.05) between the both groups was coincidental. A coefficient greater than 0.50 indicates that statistical significance is strong. Binary logistic regression-backward method was used to predict high-risk ACB, AFPS, and presence of old age (≥ 75 years).

### Ethics approval

The study was approved by the Local Ethics Committee (decision no: 2021/09–36).

## Results

### Descriptive data

Despite meeting the inclusion criteria, 41 patients who were included in the retrospective process to avoid duplication were not included in the prospective process. A total of 601 patients (64.7% female and mean±SD age of 75.10±7.25 years), including 301 patients retrospectively (control group) and 300 patients prospectively (intervention group), were included in the study. All demographic data of the patients were similar between both groups (*p*>0.05), except height, marital status and history of atrial fibrillation (*p* < 0.05). When the patients were analyzed according to the history of at least one fall within the last year, 25.6% of the control group and 31.3% of the intervention group had history of fall (Table 1).

**Table 1:** Demographic data of the patients

Variables	Control (n = 301)	Intervention (n = 300)	Total (n = 601)	*p*
**Gender, n (%)**				
Female	190 (63.1)	199 (66.3)	389 (64.7)	0.410
Male	111 (36.9)	101 (36.7)	212 (35.3)	
Age (years), mean (SD)	75.77 (7.44)	74.57 (7.01)	75.10 (7.25)	0.373
65–74	147 (48.8)	158 (52.7)	305 (50.7)	0.193
75–84	113 (37.5)	115 (38.3)	228 (37.9)	
≥85	41 (13.6)	27 (9.0)	68 (11.3)	
Weight (kg), mean (SD)	75.91 (15.23)	76.74 (14.94)	76.37 (15.06)	0.594
Height (cm), mean (SD)	162.16 (8.52)	160.56 (9.20)	161.27 (8.93)	0.034*
BMI (kg/m^2^), mean (SD)	30.33 (6.42)	29.89 (5.91)	30.09 (6.97)	0.205
**Marital status, n (%)**				
Married	184 (61.1)	185 (61.7)	369 (61.4)	0.012*
Divorced	112 (37.2)	97 (32.3)	209 (34.8)	
Widowed	5 (1.7)	18 (6.0)	23 (3.8)	
Number of children, median (range)	3 (0–9)	4 (0–9)	3 (0–9)	0.881
Smoking, n (%)	69 (22.9)	71 (23.7)	140 (23.3)	0.829
Alcohol, n (%)	5 (1.7)	9 (3.0)	14 (2.3)	0.414
Allergy, n (%)	5 (1.7)	10 (3.3)	15 (2.5)	0.293
Fall within the last year, n (%)	77 (25.6)	94 (31.3)	171 (28.5)	0.118
Influenza vaccination, n (%)	40 (13.3)	48 (16.0)	88 (14.6)	0.347
Pneumococcal vaccination, n (%)	71 (23.6)	73 (24.3)	144 (24.0)	0.831
Forgetfulness, n (%)	167 (60.5)	177 (59.0)	344 (59.7)	0.713
Progressive forgetfulness, n (%)	108 (39.3)	106 (35.3)	214 (37.2)	0.329
**Diagnosis, n (%)**				
Hypertension	233 (77.4)	246 (82.0)	479 (79.7)	0.162
Diabetes mellitus	151 (50.2)	148 (49.3)	299 (49.8)	0.838
Coronary artery disease	122 (40.5)	111 (37.0)	233 (38.8)	0.374
Hypothyroidism	43 (14.3)	35 (11.7)	78 (13.0)	0.339
Atrial fibrillation	48 (15.9)	28 (9.3)	76 (12.6)	0.015*
Osteoporosis	42 (14.0)	33 (11.0)	75 (12.5)	0.273
Asthma	42 (14.0)	33 (11.0)	75 (12.5)	0.273
Congestive heart failure	36 (12.0)	34 (11.3)	70 (11.6)	0.811
Chronic kidney disease	33 (11.0)	27 (9.0)	60 (10.0)	0.422
COPD	30 (10.0)	21 (7.0)	51 (8.5)	0.247
Acute renal failure	19 (6.3)	10 (3.3)	29 (4.8)	0.130
Liver failure	3 (1.0)	2 (0.7)	5 (0.8)	1.000
CCI, mean (SD)	6.01 (2.90)	5.50 (2.32)	5.75 (2.61)	0.559
ADLs (n = 547), mean (SD)	5.02 (1.68)	5.21 (1.43)	5.12 (1.55)	0.938
IADLs (n = 543), mean (SD)	5.92 (2.88)	6.17 (2.74)	6.05 (2.81)	0.891
MNA-SF (n = 428), mean (SD)	11.59 (3.06)	11.41 (3.52)	11.49 (3.31)	0.996
GDS (n = 454), mean (SD)	4.79 (4.33)	4.18 (3.96)	4.47 (4.15)	0.633
MMSE (n = 508), mean (SD)	25.99 (5.78)	25.43 (6.36)	25.71 (6.08)	0.379
CDT (n = 443), mean (SD)	3.85 (2.39)	3.71 (2.50)	3.78 (2.45)	0.968

BMI: Body mass index, COPD: Chronic obstructive pulmonary disease, CCI: Charlson Comorbidity Index, ADLs: Activities of Daily Living, IADLs: Instrumental Activities of Daily Living, MNA-SF: Mini Nutritional Assessment-Short Form, GDS: Geriatric Depression Scale, MMSE: Mini Mental State Examination, CDT: Clock Drawing Test

### Anticholinergic burden

Polypharmacy, which is frequently defined as the use of five or more drugs in the elderly, was observed in 84.4% of the patients in the control group and 80.3% of the patients in the intervention group. A total of 99 prescribed drugs with anticholinergic effect score were determined by clinical pharmacist using the ACBS. In this study, 34 different drugs with anticholinergic effect were identified in the control (32 drugs) and intervention (30 drugs) groups. Of these 34 drugs, 18 drugs had 1 point, 2 drugs had 2 points, and 14 drugs had 3 points. The most commonly prescribed anticholinergic drug in both groups was metoprolol with 1 point (52.2% vs. 24.6%, respectively; *p* = 0.150). All patients included in the study were prescribed a mean (SD) of 1.43 (0.72) anticholinergic drugs. The ACBS score of the intervention group was decreased from 1.72 (1.17) to 1.64 (1.08) with clinical pharmacist intervention (*p* = 0.01) (Table 2).

**Table 2:** Distribution of patients’ data on drug use

Variables	Control (n = 301)	Intervention (n = 300)	Total (n = 601)	*p*
Drugs, n (%)				
*Metoprolol* ^†^	157 (52.2)	74 (24.6)	331 (55.1)	0.150
*Furosemide* ^†^	84 (27.9)	60 (20.0)	144 (24.0)	0.023*
*Warfarin* ^†^	27 (9.0)	23 (7.7)	50 (8.3)	0.667
*Colchicin* ^†^	19 (6.3)	26 (8.7)	45 (7.5)	0.346
*Digoxin* ^†^	24 (8.0)	18 (6.0)	42 (7.0)	0.430
*Isosorbide mononitrate* ^†^	13 (4.3)	16 (5.3)	29 (4.8)	0.697
*Trazodone* ^†^	14 (4.7)	15 (5.0)	29 (4.8)	0.993
*Desloratadine* ^†^	10 (3.3)	10 (3.3)	20 (3.3)	1.000
*Cetirizine* ^†^	8 (2.7)	9 (3.0)	17 (2.8)	0.994
*Fesoterodine* ^§^	8 (2.7)	7 (2.3)	15 (2.5)	1.000
*Tolterodine* ^§^	10 (3.3)	5 (1.7)	15 (2.5)	0.299
*Solifenacin* ^§^	5 (1.7)	9 (3.0)	14 (2.3)	0.414
*Trospium* ^§^	9 (3.0)	4 (1.3)	13 (2.2)	0.262
*Paroxetine* ^§^	6 (2.0)	6 (2.0)	12 (2.0)	1.000
*Risperidone* ^†^	6 (2.0)	5 (1.7)	11 (1.8)	1.000
*Venlafaxine* ^†^	5 (1.7)	4 (1.3)	9 (1.5)	1.000
*Levocetirizine* ^†^	4 (1.3)	4 (1.3)	8 (1.3)	0.996
*Nifedipine* ^†^	3 (1.0)	3 (1.0)	6 (1.0)	1.000
*Olanzapine* ^§^	3 (1.0)	2 (0.7)	5 (0.8)	1.000
*Carbamazepine* ^‡^	3 (1.0)	2 (0.7)	5 (0.8)	1.000
*Amitriptyline* ^§^	3 (1.0)	2 (0.7)	5 (0.8)	1.000
*Hydroxyzine* ^§^	1 (0.3)	3 (1.0)	4 (0.7)	0.373
*Alprazolam* ^†^	1 (0.3)	3 (1.0)	4 (0.7)	0.373
*Captopril* ^†^	2 (0.7)	2 (0.7)	4 (0.7)	1.000
*Dimenhydrinate* ^§^	1 (0.3)	2 (0.7)	3 (0.5)	0.624
*Trifluoperazine* ^§^	2 (0.7)	1 (0.3)	3 (0.5)	1.000
*Amantadine* ^‡^	-	2 (0.7)	2 (0.3)	0.950
*Doxylamine* ^§^	1 (0.3)	1 (0.3)	2 (0.3)	1.000
*Haloperidol* ^†^	2 (0.7)	-	2 (0.3)	0.499
*Darifenacin* ^§^	-	1 (0.3)	1 (0.2)	0.499
*Oxybutynin* ^§^	1 (0.3)	-	1 (0.2)	1.000
*Clozapine* ^§^	1 (0.3)	-	1 (0.2)	1.000
*Loratadine* ^†^	1 (0.3)	-	1 (0.2)	1.000
*Diazepam* ^†^	1 (0.3)	-	1 (0.2)	1.000
Number of anticholinergic drugs, mean (SD) Median (range)	1.45 (0.72) 1 (1–5)	1.41 (0.73) 1 (1–6)	1.43 (0.72) 1 (1–6)	0.991
Total number of daily drugs, mean (SD) Median (range)	7.70 (3.18) 7 (1–21)	7.61 (3.40) 7 (1–17)	7.65 (3.39) 7 (1–21)	0.966
Polypharmacy (≥ 5), n (%)	254 (84.4)	241 (80.3)	495 (82.4)	0.193
Number of anticholinergic drugs to total number of daily drugs ratio, mean (SD) Median (range)	0.22 (0.13) 0.18 (0.06-1)	0.22 (0.45) 0.17 (0.06-1)	0.22 (0.14) 0.18 (0.06-1)	0.983
Pre-intervention ACBS, mean (SD) Median (range)	1.78 (1.17) 1 (1–9)	1.72 (1.17) 1 (1–10)	1.75 (1.17) 1 (1–10)	0.918
Pre-intervention ACBS (high risk), n (%)	66 (21.9)	62 (20.7)	128 (21.3)	0.706
Post-intervention ACBS, mean (SD) Median (range)	-	1.64 (1.08) 1 (0–10)	1.71 (1.13) 1 (0–10)	0.813
Post-intervention ACBS (high risk), n (%)	-	60 (20.0)	126 (21.0)	0.562

SD: standard deviation, ACBS: Anticholinergic Cognitive Burden Scale

According to the Anticholinergic Cognitive Burden Scale; ^†^1 point,^‡^2 points, ^§^3 points, *p < 0.05

### Aachen Falls Prevention Scale

It was found that 30% of the patients had fallen at least once within the last year and 51% of them had a fear of falling. Also, 84.7% of the patients used medication that could affect falls, and 19% had an additional disease (Parkinson’s, arthritis or rheumatism) that could affect falls. When the geriatric syndromes were examined, it was shown that 57% of the patients suffered from forgetfulness, 45.3% from incontinence, and 41.7% from depression. The median (range) value for the subjective fall risk assessment question was determined as 5 (1–10) (Table 3). The total AFPS score (mean rank: 204.82 vs. 125.71) and subjective fall risk assessment (mean rank: 190.02 vs. 132.47) were significantly higher in patients with a history of falls in the last year (*p* < 0.001).

**Table 3:** Distribution of patients’ responses to the Aachen Falls Prevention Scale (n = 300)

Items	Yes, n (%)	No, n (%)
*Part I*		
Do you have problems with hearing or vision?	138 (46.0)	162 (54.0)
Do you feel unsafe or have you been falling recently?	90 (30.0)	210 (70.0)
Are you afraid of falling?	153 (51.0)	147 (49.0)
Do you take medication for sleep, cardiac problems, diuretics, or sedatives?	254 (84.7)	46 (15.3)
Do you loose urine or stool involuntarily?	136 (45.3)	164 (54.7)
Do you have memory problems?	171 (57.0)	129 (43.0)
Do you feel lonely at times and think that your life is without value?	125 (41.7)	175 (58.3)
Do you use a walking aid on a regular basis?	101 (33.7)	199 (66.3)
Do you suffer from Parkinson’s, Arthritis or Rheumatism?	57 (19.0)	243 (95.3)
Are there many traps that might cause a fall in your home?	14 (4.7)	286 (95.3)
**Total score, median (range)**	**4 (1–9)**	
*Part II*		
Stand freely, do not lean or hold on anybody, measure the time until you have to do a corrective action with your arm, upper body or lower extremity (Successfully completed: 20 s or more, Failed: less than 20 s)	229 (76.3)	71 (23.7)
How would you grade your falls risk on a scale of 1 to 10 (10 … max. risk)? median (range)	5 (1–10)	

Patients with history of fall gave themselves higher scores [5.54 (2.20)] in subjective fall risk assessment than patients without any history of fall [3.92 (2.46)] (*p* < 0.001). When the high-risk (n = 128) and low-risk (n = 172) patients categorized according to the AFPS were compared in terms of co-morbidities, the prevalence of osteoporosis was significantly higher in high-risk fall group (*p* = 0.017). While 93.6% of the patients in the low-risk group were able to successfully complete the standing test, this rate was 53.1% in the high-risk group (*p* < 0.001). Also, it was found that the performance for comprehensive geriatric assessment tests [(Activities of Daily Living (ADLs), Instrumental Activities of Daily Living (IADLs), Mini Nutritional Assessment-Short Form (MNA-SF), Geriatric Depression Scale (GDS), Mini Mental State Examination (MMSE), and Clock Drawing Test (CDT)] were significantly worse in the high-risk group than in the low-risk group according to the AFPS (*p* < 0.001).

When binary logistic regression analysis was performed to determine the surrogate outcome of AFPS while assessing falls, each 1-point increase in the total AFPS score increases the odds of a history of falls within the last year by 1.726 times (*p* < 0.001).

The mean anticholinergic burden of the high and low risk groups according to the AFPS was compared in the pre- and post-intervention periods. A significant difference was found between the high and low risk groups in terms of anticholinergic burden in both the pre- and post-intervention periods (*p* = 0.035 and *p* = 0.013, respectively). According to ACBS, 24.2% of high-risk (≥ 3 points) patients had a high fall risk, while 18.0% of low-risk patients had a high fall risk.

When binary logistic regression analysis was performed to determine the factors predicting falls, the risk of falls was 2.503 times higher in high-risk patients according to the ACBS (*p* = 0.034). Each 1-point decrease in the IADLSs increases the risk of falling by 1.207 times (*p* = 0.002).

When binary logistic regression analysis was performed to determine the factors predicting high-risk ACB, a history of falls within the last year and osteoporosis increase the odds of having a high-risk ACB by 2.026 (*p* = 0.033) and 3.239 times (*p* = 0.003), respectively. In addition, ACB was found to be more common among patients over the age of 75 in our study (24.3% vs. 18.4%). Patients with middle-oldest-old ages (≥ 75 years; n = 296) were 2.055 times at higher risk for falling than patients with youngest-old ages (< 75 years; n = 305) (*p* < 0.001).

### Clinical pharmacist intervention

The clinical pharmacist made 19 recommendations to reduce the ACB of 16 patients in the intervention group, of which 17 (89.47%) were accepted by the geriatricians.

Accepted recommendations;

- Discontinuation of desloratadine (3 patients), cetirizine (3 patients), levocetirizine (2 patients), doxylamine, hydroxyzine, alprazolam, solifenacin, trospium, trazodone, fesoterodine.
- Switching of paroxetine with any selective serotonin reuptake inhibitor (2 patients).

Rejected recommendations;

- Discontinuation of risperidone due to moderate drug-drug interaction between mirtazapine and risperidone.
- Discontinuation of fesoterodine.

## Discussion

We conducted this study in a medical center with a geriatric outpatient clinic to retrospectively assess patients’ anticholinergic burden with ACBS and to prospectively assess patients’ fall risk with AFPS to evaluate the effect of clinical pharmacist interventions. We also aimed to examine the effect of patients’ demographic and clinical parameters and other routine assessments on ACBS and AFPS.

ACBS was preferred due to its specificity for cognitive functions. According to a systematic review, the analysis of citations for individual scales indicated that the ACBS emerged as the most frequently validated expert-based anticholinergic scale concerning adverse outcomes [17]. In another systematic review that systematically compared all anticholinergic scales and evaluated their association with clinical outcomes, ACBS demonstrated the highest quality percentage in terms of applicability and one of the most investigated clinical outcomes, falls, have yielded conflicting results [25].

While the answers to the questions in the AFPS are purely subjective and based on patient self-report, the medications in the ACB are purely objective and derived from patient prescriptions and/or computerized physician order system. Therefore, drug-related data obtained from the ACB are more reliable. In addition, while the general statement in the AFPS represents a broad group of anticholinergic drugs, the ACB examined in detail whether each drug was prescribed or not by the clinicians.

The fact that the ACB of the most prescribed drugs in our study was 1 point (metoprolol, furosemide, warfarin, etc.) should not be ignored. In older patients with polypharmacy, the risk of anticholinergic effects increases with cumulative exposure to these drugs which may be neglected by geriatricians [24]. Similarly, in a study, drugs for patients of ACB scores were most commonly treated with the cardiovascular system drugs (such as metoprolol and nifedipine) [26]. In another study, one of the most frequently prescribed drugs with anticholinergic burden was furosemide [27]. The concomitant use of different drugs by older adults contributes significantly to the occurrence of adverse drug reactions (ADRs). The risk of ADRs is expected to increase by roughly 50% with the use of 5 medications and surpasses 95% with the use of more than 8 drugs [28]. Although polypharmacy and ACB are distinct concepts, they are known to be closely related, with ACB usually accompanying polypharmacy [29]. Polypharmacy (≥ 5 drugs) was higher in the control group than in the intervention group in our study, which was higher than in previous studies. It has been observed that, similar to our study, polypharmacy is not a risk factor for falls, but high-risk anticholinergic medication use is [30]. There have also been studies that show that chronic conditions are a better predictor of falls than polypharmacy [31]. According to the literature, one-third of patients over 75 and 49% of patients over 85 are prescribed at least one anticholinergic medicine [27, 32]. Similarly, ACB was found to be more common among patients over the age of 75 in our study.

Although it is not certain that every increase in ACB will increase the risk of ADRs, there are a number of advantages to not providing medications with anticholinergic effects to older adults. Deprescribing can reverse ADRs as well as prevent issues like fall risks [33]. According to a prevalence study, over a 20-year period, the use of strong anticholinergic drugs nearly doubled among older adults living in England, with some of the biggest increases occurring among those who are most susceptible to anticholinergic ADRs [34].

According to Neal et al. [35], whereas ACB score of 2 or higher increased the likelihood of recurrent falls by 2.54 times (*p* = 0.004), no relationship between increased ACB and the prevalence of osteoporosis. On the other hand, in our study, a history of fall and a diagnosis of osteoporosis were independent risk factors for having a high-risk ACB.

In our study, falls were shown to be associated with ACB, whereas one study [36] showed an association with the number of anticholinergic drugs and duration of treatment, and another study [37] showed an association with continuous use of anticholinergic drugs.

The direct effect of polypharmacy on falls could not be demonstrated both in our study and in the study by Ziere et al. [30]. In contrast to the study by Neal et al. [35], we found that the presence of osteoporosis in the patient increased the likelihood of high ACB by 3.239 times. The fact that 16.4% of patients with high-risk scores and 7% of patients with low-risk scores had osteoporosis (*p* = 0.017) indicates that patients with osteoporosis are more prone to falls. However, a stronger association could not be established due to the small number of patients with osteoporosis. Our study also showed that high-risk anticholinergic drug use was an important factor on the risk of falls (*p* = 0.034). Therefore, the importance and threshold value of ACB rather than the number of drugs were found to be more accurate and meaningful.

The current literature [24, 35, 37] revealed that falls were solely determined by questioning patients, “Have you fallen in the last 12 months?” In addition to this question, the AFPS was utilized in our study to assess non-drug-related problems that may cause falls and to create a quantitative score for falls in response to the potential of patients forgetting their falls within the last year. In our study, we found that the risk and fear of recurrent fall were higher in patients with a history of falls within the last year. Similarly, Wapp et al. found the number of previous falls and fear of falling to be predictive of a personalized fall rate estimate for community-dwelling older adults [38].

The IADLs score reduced considerably in the group with falls (*p* < 0.001), according to a study [39]. In our study, we discovered a negative relationship between IADLs and falls. Each one-point decline in this score is associated with a 20.7% increase in falls (*p* = 0.002).

The recommendations offered to geriatricians who are aware that recommendations are offered within the framework of the study are based entirely on evidence and current literature. To prevent bias and ensure the independence of the recommendations, the geriatricians participating in the study conducting group differ from those involved in the intervention provider group.

Studies showing that clinical pharmacist intervention reduces inappropriate anticholinergic drug use [40]. As a result of the clinical pharmacist intervention, the ACB score decreased by 2 points (*p* < 0.001) and remained unchanged in the control group [41]. Similarly, in our study, the mean ACBS was reduced from 1.72 to 1.64 with clinical pharmacist intervention, while it remained unchanged in the control group (*p* = 0.01). While the presence or intervention of a clinical pharmacist does not have a significant effect on the mean number of anticholinergic drugs, a significant decrease in the mean ACBS is due to the high score (2 or 3 points) of the accepted recommendations. Despite the small number of patients and interventions, it is estimated that not only the clinical pharmacist recommendations but also the presence of the clinical pharmacist in geriatrics had an impact on the significantly decrease in the mean ACBS with Hawthorne effect which refers to the change in behavior of clinicians when they are monitored more closely [42]. Also, since inpatients were not included in the study, patients were regularly followed up in the outpatient clinics by geriatricians who were experts in drug-related problems, and the clinical pharmacist evaluated the patient after the geriatricians, therefore the intervention of the clinical pharmacist to reduce ACB was limited due to the geriatricians’ prioritized intervention on ACB.

Other limitations of the study are that only one interview was conducted in a limited period of time and only continuously used drugs (except in case of need) were taken into consideration without assessment of medication adherence. Also, we did not evaluate the effect of long and short-term cumulative exposure to anticholinergic drugs due to retrospective process. We acknowledge that one of the limitations of the study is that we used the ACB developed by Boustani 15 years ago. However, the updated version in 2012 after a review of new information and newly approved medications, was used in this study. Therefore, we preferred it because it is one of the most cited and well-known scales in the current literature.

## Conclusion

In this study, it was found that the risk of falls in older adults with high-risk ACB is 2.5-fold increased. It was also shown that clinical pharmacist intervention was effective in reducing ACB. Due to the high rate of ACB in the older adults and the widespread use of risky drugs in terms of anticholinergic effect, ACB of drugs should be taken into account during prescribing and the intervention of the clinical pharmacist in detecting, managing and reducing ACB should be taken into consideration.

## Data Availability

The data presented in this study are available on request from the corresponding author. The data are not publicly available due to restrictions privacy and ethical.
